# Ethics at the Centre of Global and Local Challenges: Thoughts on the Future of Business Ethics

**DOI:** 10.1007/s10551-022-05239-2

**Published:** 2022-10-05

**Authors:** Steffen Böhm, Michal Carrington, Nelarine Cornelius, Boudewijn de Bruin, Michelle Greenwood, Louise Hassan, Tanusree Jain, Charlotte Karam, Arno Kourula, Laurence Romani, Suhaib Riaz, Deirdre Shaw

**Affiliations:** 1 Department of Sustainable Futures, University of Exeter Business School, Exeter, UK; 2 Faculty of Business and Economics, University of Melbourne, Melbourne, Australia; 3 Queen Mary, University of London, London, UK; 4 Faculty of Economics and Business, University of Groningen, Groningen, Netherlands; 5 Faculty of Philosophy, University of Groningen, Groningen, Netherlands; 6 Department of Management, Monash University, Melbourne, Australia; 7 Department of Marketing, University of Birmingham, Birmingham, UK; 8 Department of Management, Society and Communication, Copenhagen Business School, Frederiksberg, Denmark; 9 University of Ottawa, Ottawa, Canada; 10 American University of Beirut, Beirut, Lebanon; 11 University of Amsterdam Business School, Amsterdam, Netherlands; 12 Department of Management and Organization, Stockholm School of Economics, Stockholm, Sweden; 13 Telfer School of Management, University of Ottawa, Ottawa, Canada; 14 Adam Smith Business, University of Glasgow, Glasgow, UK

**Keywords:** Grand challenges, Climate change, Consumer ethics, Cultural differences, Inequality, Capability approach, Feminism

## Abstract

To commemorate 40 years since the founding of the Journal of Business Ethics, the editors in chief of the journal have invited the editors to provide commentaries on the future of business ethics. This essay comprises a selection of commentaries aimed at creating dialogue around the theme *Ethics at the centre of global and local challenges*. For much of the history of the Journal of Business Ethics, ethics was seen within the academy as a peripheral aspect of business. However, in recent years, the stakes have risen dramatically, with global and local worlds destabilized by financial crisis, climate change, internet technologies and artificial intelligence, and global health crises. The authors of these commentaries address these grand challenges by placing business ethics at their centre. What if all grand challenges were framed as grand *ethical* challenges? Tanusree Jain, Arno Kourula and Suhaib Riaz posit that an ethical lens allows for a humble response, in which those with greater capacity take greater responsibility but remain inclusive and cognizant of different voices and experiences. Focussing on business ethics in connection to the grand(est) challenge of environmental emergencies, Steffen Böhm introduces the deceptively simple yet radical position that business *is* nature, and nature *is* business. His quick but profound side-step from arguments against human–nature dualism to an ontological undoing of the business–nature dichotomy should have all business ethics scholars rethinking their “business and society” assumptions. Also, singularly concerned with the climate emergency, Boudewijn de Bruin posits a scenario where, 40 years from now, our field will be evaluated by its ability to have helped humanity emerge from this emergency. He contends that *Milieudefensie (Friends of the Earth)* v. *Royal Dutch Shell* illustrates how human rights take centre stage in climate change litigation, and how business ethics enters the courtroom. From a consumer ethics perspective, Deirdre Shaw, Michal Carrington and Louise Hassan argue that ecologically sustainable and socially just marketplace systems demand cultural change, a reconsideration of future interpretations of “consumer society”, a challenge to the dominant “growth logic” and stimulation of alternative ways to address our consumption needs. Still concerned with global issues, but turning attention to social inequalities, Nelarine Cornelius links the capability approach (CA) to global and corporate governance, arguing that CA will continue to lie at the foundation of human development policy, and, increasingly, CSR and corporate governance. Continuing debate on the grand challenges associated with justice and equality, Laurence Romani identifies a significant shift in the centrality of business ethics in debates on managing (cultural) differences, positing that dialogue between diversity management and international management can ground future debate in business ethics. Finally, the essay concludes with a commentary by Charlotte Karam and Michelle Greenwood on the possibilities of feminist-inspired theories, methods, and positionality for many spheres of business ethics, not least stakeholder theory, to broaden and deepen its capacity for nuance, responsiveness, and transformation. In the words of our commentators, grand challenges must be addressed urgently, and the Journal of Business Ethics should be at the forefront of tackling them.

## Reimagining Grand Challenges as Grand Ethical Challenges: From Grandiose to Humble Solutions^1^

**Tanusree Jain, Arno Kourula and Suhaib Riaz**

**Introduction**

“The issues we face are so big and the targets are so challenging that we cannot do it alone, so there is a certain humility and a recognition that we need to invite other people in. When you look at any issue, such as food or water scarcity, it is very clear that no individual institution, government or company can provide the solution”.—Paul Polman, Former CEO of Unilever (Confino, 2012).

“According to the constitution indigenous peoples … should be included in these decisions. But in practice this hasn’t occurred. The neoliberal sectors of our country have followed policies of the IMF and InterAmerican Development Bank (IDB)”—Ross Mary Borja, EkoRural, Ecuador (Groundswell International, 2021).

In this essay, we examine the questions of what it means to look at grand challenges from an ethical lens and why it is important. Within management scholarship, grand challenges are understood as “formulations of global problems that can be plausibly addressed through coordinated and collaborative effort” (George et al., 2016, p. 1880). Scholars have described grand challenges as complex, uncertain and evaluative (Ferraro et al., 2015). In other words, although grand challenges are issues that transcend national boundaries and are global in nature and scope, they often involve disjointed dynamics in terms of their non-linearity, they embed emergent understandings, and they result in multiple experiences in terms of their impact on different parts of the human and non-human world. While the notion of grand challenges has recently started receiving fairly extensive attention within management scholarship, the power imbalances inherent in framings, actors, and contexts have somewhat skewed our understanding of them. These power imbalances revolve around issues of transparency, decision making and participation: Who can propose solutions and approaches and who cannot (due to either resource-constraints or structural marginalization)? Who is heard and who is silenced? Through this commemorative issue of the Journal of Business Ethics, we discuss and highlight how business ethics lenses allow us to reimagine grand challenges and think of them as “grand ethical challenges”. Our aim is to reflect on the role of “humble solutions” to resolve them.

**Fig. 1:**
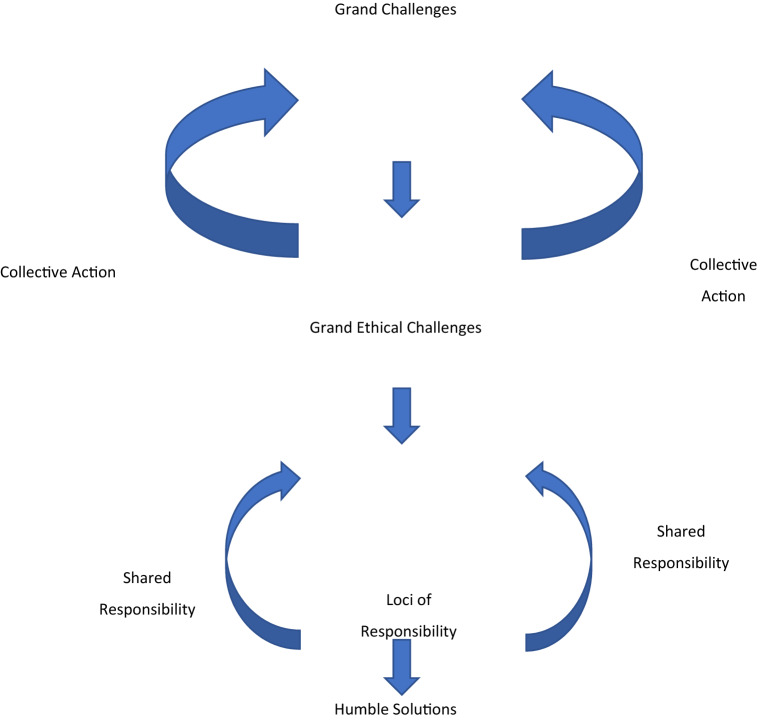
From grand challenges to humble solutions

The key arguments of our essay in a conceptual framework are presented in Fig. 1. We begin by presenting the extant view on grand challenges and argue for adopting an ethical lens to examine them. We propose that infusing grand challenges with ethics allows us to redefine them as *Grand Ethical Challenges*. Using examples, we show how the reimagining with an ethics lens can allow us to see the challenge in a different light or from a different perspective. Next, we discuss how grand ethical challenges necessitate a review and revision of the loci of responsibility for these challenges. All grand challenges are embedded within a certain way of seeing and assigning responsibility, and ethics pushes us to question these ways. Ultimately, the discussion of the loci of responsibility is closely connected to the search for solutions. We argue that the solutions to challenges that are often described in a grandiose way do not necessarily have to be so. We call for *humble solutions* to grand ethical challenges––a core element of this humility is being inclusive and cognizant of different voices and experiences in and around grand ethical challenges. These humble solutions allow for shared responsibility taking and can lead to collective action to address grand challenges.

**From Grand Challenges to Grand Ethical Challenges**

At the outset, we suggest that business ethics allows us to look at grand challenges differently. Ethical analysis aims to go to the (philosophical) roots, including historical normative understandings of related concepts and frameworks. The field of business ethics (exemplified by numerous *Journal of Business Ethics* articles) has been foundational in focusing on actors beyond the large multi-national firms and western contexts, often looking at business from outside of business itself. In this manner, business ethics has consistently been able to problematize dominant discourses. The aim of the field is not just measuring impact of stakeholders on firm-level outcomes, but also the reverse, i.e. the impact of firms and of non-business organizations on stakeholders and society. With a strong interest in fairness and justice-based perspectives, ethics is central to examining which perspectives should be explored and whose interests are served. This helps to evaluate who deserves to be heard in a particular situation for moral reasons. By going deeper in this respect, utilizing ethics lenses can support the reimagination of grand challenges.

Management practice has so far sought silver bullet (often technological) solutions to grand challenges. We propose that an ethical analysis will offer a different, more inclusive, perspective on grand challenges, one that can extend to include marginalized groups and even non-human stakeholders. For example, while a management approach to climate change might focus on strategies for emissions reductions and setting science-based targets, an ethical approach could focus on global climate justice. This includes examining the responsibility of different actors, both currently and historically, and especially focus on those most affected and marginalized in climate negotiations. Climate change through ethical lenses involves a debate of appropriate moral responses (see Romar, 2009). While employees and the general public may view new technologies such as artificial intelligence (AI) and machine learning as threatening employment, ethical analysis would typically explore this theme in the broader context of changing notions of meaningful work and ethics in human–machine interaction. This involves acknowledging that meaningful work is inherently a normative concept (Michaelson, 2021) and the moral foundations of AI need to be so evaluated (Telkamp & Anderson, 2022). While management scholars may identify climate change and poverty as grand challenges that need large-scale interventions of governments and corporations for developing solutions, business ethicists might re-examine and problematize grand challenges in a manner that re-conceptualizes the relationship between humans and nature (see Tallberg et al., 2021) and identifies the marginalization inherent in who gets to sit at the table where “solutions” are explored (e.g. local communities and grassroots organizations). Ultimately, grand ethical challenges such as those of inequality and biodiversity should be seen as intertwined instead of as separate problems to address.

***The Loci of Responsibility***

To untangle issues of ethics in grand challenges and redefine them as grand ethical challenges, we bring to the fore the importance of responsibility vis-à-vis grand challenges. While there is some recognition that there are varying levels and types of responsibilities that social actors have in how they are implicated in these grand challenges, an in-depth and systematic discussion of responsibilities is not yet sufficiently developed. We follow the view that, in current times, “obligations of justice extend globally”, because “structural social processes connect people across the world without regard to political boundaries” (Young, 2006, p. 102). Furthermore, these obligations extend to all social actors but “those institutionally and materially situated to be able to do more to affect the conditions of vulnerability have greater obligations” (Young, 2006, p. 106). In other words, we need to discuss the loci of responsibility—what scale and scope of responsibility should be attributed to various actors in grand challenges. For example, where lies the responsibility for deforestation of a local area in a poor country of the Global South that is tied to the grand ecological challenge of biodiversity loss? There may be local loci of responsibility such as local leaders or communities that may not have considered alternatives; but there may also be systemic pressures at the national level that make alternatives too costly or unviable; and these in turn may be tied to global economic and political systems from where such pressures cascade downwards. Each one of these would constitute a relevant locus of responsibility. Opening up a discussion of fairness and justice in considering responsibility for grand challenges would aid us in understanding them as grand ethical challenges.

There are several more terrains to tread here, because we already know how closely local, national and global issues are interconnected. For example, the grand challenge of economic inequality may have multiple facets such as inequality between poor migrant workers and others in a city, which may be rooted in wider urban–rural inequality within a country, and which in turn may be tied to global production networks or how work is distributed and outsourced across the globe. There are many local and global actors, including MNEs and governments, that are implicated in such a challenge. Furthermore, the inequality challenge may be perceived quite differently, based on the vantage point adopted—for example, research shows that, while global inequality has reduced, national-level inequality has increased. The question of who is responsible for inequality at one level or in one location is likely to be a vexing and complex one, but asking this question does bring an ethics discussion to the fore.

This agenda is indeed touched upon in research dispersed across several areas. For example, moral disengagement on the issue of worker’s rights has been tied to where a firm’s “responsibility boundaries” are placed across a global value chain (Egels-Zanden, 2017). Scholars have highlighted how large fossil fuel companies use mythmaking to retreat to past comfortable positions, escape harsh realities, and shift blame to others, in effect finding ways to avoid responsibility for climate change (Ferns et al., 2019). Similarly, a large beverage multi-national, Coca Cola, projects responsibility for the social issue of obesity outside of the organization in an attempt to absolve itself of ethical lapses (Iivonen, 2018). While such studies show the potential for work in this area, more theoretical and empirical development is needed on loci of responsibility to infuse ethics in the research on grand challenges. Importantly, our view of responsibility has to go beyond just identifying liability to also finding ways of assigning social roles or positions towards achieving outcomes (Young, 2006). We now turn to this aspect.

**From Grandiose to Humble Solutions**

The loci of responsibility is a useful lens that can help us to not only problematize the grand challenges more accurately in terms of actors implicated, but also help to conceptualize solutions and actions that rely on ethical reasoning. To date the solutions to grand challenges have been approached by placing the loci of responsibility on governments and states, on the one hand, and large corporate actors, on the other. This is because the access to resources, the strength of capabilities, and the depth of networks needed to deploy the former reside fundamentally within these two entities. In parallel, the emergence of multi-stakeholder initiatives (MSIs) to complement the efforts of governments is also not new in the quest to harness grand solutions.

Interestingly, and indeed unfortunately, we have observed the endless and often unfruitful discussions on fixing responsibility for climate change. For example, if there is to be carbon tax or other similar measures, what should be the role of social actors most responsible for the climate change challenge and who should share and thereby contribute most to the burden in this solution often remains under-explored. Equally, despite the increasing prevalence of transformative business models and MSIs, neither actors in the political arena nor those in the economic arena appear to be synchronized in their motivations, narrative, and action towards devising solutions for grand challenges (Dentoni et al., 2018). The lack of coordinated efforts in this domain can lead to grave multi-fold impacts on the process of devising urgent solutions.

First, it is likely to result in disengagement towards grand challenges by the very actors on which humanity places faith and loci of responsibility in finding solutions. For example, in the aftermath of the Paris Climate Agreement, several companies in the Tech, Oil and Gas and Automobile sector have announced climate pledges in the form of becoming carbon negative or achieving “net-zero” status in the next couple of decades. Yet regulations on corporate disclosure on climate action are still soft, and lack of benchmarks makes it difficult to compare corporate action and progress on climate change. Both signal potential lack of genuine engagement on the part of powerful actors in tackling grand challenges.

Second, the focus on state, MSI, and corporate efforts on grand challenges creates disempowerment among a large section of stakeholders, whose voices remain unheard in the process of governance for grand challenges. Although large-scale technological transformations are essential for tackling grand challenges, persistent disparities within and across communities require differentiated solutions. When those who are affected––such as indigenous communities, marginalized farmers, and migratory workers––are consistently out of the dialogue and solution building space, it results in a sense of loss akin to disenfranchisement.

And third, uncoordinated efforts create fatigue among the general public by emphasizing the sheer formidability and emergent nature of these challenges, on the one hand, and the long-term futuristic narrative weaved within proposed solutions, on the other. This negatively impacts present time creative endeavours on the part of large actors and results in kicking the can down the road into the future, while also unintentionally under-appreciating the capabilities of local communities, small and medium enterprises, and their collective cultural experiences in joining the process of governance for grand challenges and finding pragmatic sustainable solutions.

In this sense, the conceptualization of grand challenges into grand ethical challenges allows for *new ways of seeing*, *new ways of exploring* and *new ways of learning* (Friedland & Jain, 2022). We argue that it is important to look beyond the states and the big corporations as the focal points in the process of governance and finding solutions. Involvement of multiple actors and contexts will bring to the fore the need to listen humbly, and thus inclusively, and complement existing solutions by an ethically grounded contextual exploration and experimentation. For example, Tuazon et al. (2021) suggest that, to tackle the challenge of freshwater management, an organizational perspective-taking approach is needed to comprehend the complexity of the problem and to facilitate decision making. Here, they identify several actors and voices, such as researchers and academics, media and citizen response groups as key, beyond the government and the private sector. It is also noted that such a perspective-taking approach results in revealing that the grand challenge of freshwater management is perceived differently by the different actors, directly impacting the perception of progress on the challenge itself. For instance, while private businesses were found to be primarily concerned about their own business sustainability, as opposed to sustainability of the environment, for local government bodies, freshwater preservation was an economic goal to preserve livelihood and sustain growth. In contrast, for local communities freshwater preservation was a part of their cultural heritage and value system, sometimes even over and above livelihood sustenance. Here, differences in perspective on freshwater management can emerge between upstream and downstream communities.

Adding the multi-actor analysis to a multi-contextual analysis of inequality, Di Lorenzo and Scarlata (2019) suggest how inequality, as a grand challenge, may have macro-level consequences, but as a grand ethical challenge it is tied to the local context and shapes micro-level behaviours. In their view, solving such a challenge requires participation of local social enterprises that understand the problem and it requires interaction and support of institutional actors to help resolve them collectively and with scale. Indeed, frugal and social innovations are often mentioned as social and economically inclusive ways of innovating to solve grand challenges. However, the challenges of scale facing such initiatives are well known. We argue that, instead of placing major responsibility on such actors and absolving other larger actors, an ethically driven approach would instead consider multiple voices and perspectives in the process of governing and finding solutions. Local actors are repositories of knowledge through their learnings, failures, and successes. As such, listening to these actors with humility could help us understand the local manifestations of grand challenges on the ground, while also bringing attention to the systemic-level problems that stall their local solutions. It can help break the deadlock that currently prevents the generation of solutions and also overcome a sense of helplessness that pervades discussions of how to move forward on grand challenges. Accordingly, the inputs of local actors could be crucial in envisioning and assigning responsibility across a varied set of actors by considering wider loci of responsibility. This approach, we argue, is fundamentally built on the ethical principles of fairness and justice, as opposed to an ad hoc role postulation or use of power dynamics for solution building. We call for a systematic and collective effort on the part of business ethics scholars to broaden, deepen and give new energy to this agenda by infusing ethics in grand challenges through a consideration of loci of responsibility and accordingly push us towards ethical, pragmatic and inclusive solutions.

**Conclusion**

In this essay, we make three key arguments. First, we add to the developing literature in management research on grand challenges by arguing that a helpful line of inquiry is to reimagine grand challenges as grand ethical challenges. Second, we point out how this reimagining can be aided by a renewed discussion about the loci of responsibility of actors implicated in grand challenges. Third, we argue that these loci of responsibility discussion can lead to humble solutions that are ethically and practically grounded, which in turn can facilitate processes of taking shared responsibility and collective action to address grand challenges.

## Business Ethics in the Age of Climate and Ecological Emergencies

**Steffen Böhm**

**Introduction**

Global climate change is arguably the biggest challenge humanity has ever faced. Given the speed of changes to the climate already recorded, one can predict that the remainder of the twenty-first Century will be dominated by increasingly desperate attempts to radically curb greenhouse gas emissions, as well as adapt to changes that can no longer be avoided. While the climate receives most of the headlines, global warming is embedded in much wider ecological emergencies (e.g. ocean acidification, deforestation, water pollution, species extinction, soil erosion, and degradation) that are sometimes forgotten or ignored. While the Journal of Business Ethics has done more than most business and management journals to debate these challenges, much more is needed if we want to stay relevant. Nothing less, I argue, than a fundamental rethink of the relations between business, society, and nature is required to account for the environmental emergencies faced in the 21^st^ Century. Here, I will outline how such a rethink might look like, hopefully inspiring business ethics scholars to take up some of these agendas in their future research.

**Business is Nature**

When the relationship between business and nature (and business and society, for that matter) is considered, it is most common to read statements such as: “Company X has successfully reduced its greenhouse gas emissions by X per cent but has to do a lot more”; or “Company Y has successfully reduced its use of single-use plastic packaging to make a positive contribution to global waste reduction efforts”; or “Company Z is the worst offender when it comes to its destructive impact on ecosystems”. All of these utterances arguably depend on quite a crude, dualistic understanding of the relationship between business and nature, as if one can separate the two. Let me be clear: business *is* nature, and nature *is* business. I can see some environmentalists’ toenails curl up when reading such an assertion, given that the view that nature needs protecting and conserving is so entrenched in modern society, particularly in the Global North. So let me explain.

There is now a growing realization that there is a need to go beyond dualistic conceptions of society–nature relations. All living beings, including humans, are part of the wider planetary system that we call Earth. “We are all compost”, as Haraway (2015) calls it. We are “compost” in a system that has always changed and will continue to do so. It is a self-regulating, complex system with millions of nodes and feedback loops between them—Lovelock (2016) calls this “Gaia”. Geological “disasters”, such as millions of volcanic eruptions, emitting gigatons of CO_2_ into the atmosphere, made life possible on this planet in the first place. Having billions of humans on this planet who all breathe, eat and excrete has already altered this planetary system and will continue to do so, not to mention the billions of farm animals whose flatulence results in emitting gigatons of methane into the atmosphere. This has popularly been referred to as the Anthropocene, the current geological epoch during which humanity’s dominant position on Earth has already altered its geology and planetary systems.

However, a non-dualist systems perspective also requires us to look at the specificity of how humanity relates to nature. Hence, a system approach does not invite the ludicrous assumption that, because everything is fuzzy and complex, we cannot analyse and critique what is going on. Ethical theory is precisely about that: to develop normative frames of understanding of what the good life should look like. In this regard, it is perhaps more apt to call the current epoch “capitalocene”—although see Haraway’s (2015) problem with “big words”—given that the planet has been dominated by a very specific political-economic-cultural-ecological system that we call “capitalism” for at least 250 years now. Ecological world-systems theorists such as Moore (2015) have eloquently shown that capitalism has developed the way it has because of a particular way it relates to nature. Capitalism, he argues, is an ecological regime precisely because it has successfully made nature work for its own ends, altering it along the way. It is for this reason that every single business is always already an ecological business, regardless of whether it sells locally produced, vegan food wrapped in decomposable packaging or extracts oil from the ground. Business should be understood as a node of a complex ecological system, rather than simply as an entity that impacts on nature “out there”. Developing such a non-dualistic understanding of business and nature is precisely the task I see as urgent for business ethics.

**Green Solutionism**

It is not radical or controversial anymore to say that human activity is fundamentally altering the Earth system. The whole world is talking about climate change, which we know is real and already happening. The Bangladeshi peasant farmer, whose land is slowing sinking into the sea, knows it. The German small business owner, who lost their existence in the recent floods there, also knows it. Concern and attribution normally stop with climate change. Yet (social) media channels are also full of details of the multiple ecological crises we face: businesses extract billions of tons of minerals out of the ground, often in environmentally very damaging ways; the oceans are littered by millions of tons of plastics; modern agriculture, transport structures, and urbanization have displaced wildlife to the fringes, causing it to face, in many cases, extinction. The list of environmental crises is long, but so is the list of solutions offered to address them.

Businesses are now often falling over each other to declare carbon net-zero commitments, install renewable energy, invest in ESG funds, publish CSR and sustainability reports, reduce waste in their supply chains, encourage their staff and customers to eat vegan or vegetarian food, determine their carbon footprint and then offset their emissions, sell “carbon neutral” products and services (or “climate positive” or “carbon negative”—whatever the chosen speech act), among many other business solutions offered. All this is normally accompanied by glossy, multi-channel advertising campaigns that portray business as being “part of the solution”, as the saying goes. Green business is big business these days. It is becoming normalized—and this makes perfect sense. As the crises get bigger and multiply, public concern rises, and hence business needs to align itself with that concern. A business’s social licence to operate can evaporate overnight. Hence business needs to engage in a range of strategic communication to make sure it stays relevant and on the “right side of the debate”.

There is a need to critically scrutinize the multiple solutions offered by businesses—but also by governments, NGOs and multi-stakeholder governance initiatives—to tackle climate change and the many other ecological crises we face. As I said, celebrating these green solutions is not enough anymore. In an era of ecological breakdown and intense public concern over such issues, all businesses, governments, and civil society actors need to be seen to be green. The question is whether the offered solutions add up to anything significant or whether they are part of an ever-increasing cycle of greenwashing. Let me now outline some of the key questions business ethics scholars should ask, from my point of view, in order to critically scrutinize the green solutions offered by business around the world.

**Cyborgs**

After more than 50 years of post-structural and post-humanist thinking, it is surprising that most of business ethics scholarship is still dominated by quite a crude anthropocentrism. One would have thought that planetary crises, such as climate change, would have given rise to a new understanding of the relationship between business, society, and Earth’s ecosystems. While fantastic new ways of understanding contemporary crises have emerged, most business ethics scholarship still assumes that *homo sapiens* should be given a privileged role in our understanding of how ecological systems function.

Of course, humans matter. They matter immensely. But perhaps they should be understood as cyborgs (Haraway, 1991)—a cybernetic organism, which is a hybrid, some “thing” between a machine and an organism. It is human and non-human. It is a product of complex relations of ever-changing human and non-human interactions. Humans have invented words such as “woman”, “mouse”, “dog”, “house”, “green capitalism”, “net-zero”—but perhaps they are simply symbolic registers for a complex set of human–non-human relations that are non-essentialist, ever-changing and self-organized.

“Self-organized”, here, means that these human–non-human, cybernetic systems might be best understood as autopoietic (Maturana & Varela, 1980), which means that they are self-referential and self-reproducing. An autopoietic system is thus a network of processes that are not controlled by one privileged actor, the human, but, instead, by a complex web of interactions between components (or actants) that continuously regenerate the very network that produced them in the first place. The relations are constitutive, meaning that they produce concrete things in specific places, which cannot be, again, controlled by one actant alone. If one aspect of the network changes, it changes the whole network. If that change is rapid and sustained, then the whole network may create a tipping point, transforming, or rather flipping over, into a new state.

**Ontologies**

If we talk about networks of autopoietic relations, then it is clear that there is not only ONE way of being in the world. Yet the field of business ethics is still very narrow, as it is dominated by Western, Global Northern scholars who have been trained in understanding business–society–nature relations in specific ways, often ignoring other ways of being. There is an urgent need for the field of business ethics to acknowledge and engage with different ontologies and cosmologies that think about and practise nature–human interactions in a variety of different ways, challenging dominant Western paradigms.

The world is actually a “pluriverse” of many different worlds (Escobar, 2020). It is time that business ethics gave much more voice to different ways of being and existence. Indigenous communities and ecovillage communities are examples of “ecocultures” that live life differently (Böhm et al., 2014). Their ontological assumptions about what it means to live—and even what it means to do business—are very different from the dominant way of the world. While it is easy to romanticize such communities, it is nevertheless important to acknowledge their different ways of being (Ehrnström-Fuentes, 2016).

This recognition of the Other is a political and ethical act in its own right (de la Cadena & Blaser, 2018). In this way, an ontological approach to understanding business ethics is always already political and needs to be understood in this way. For example, understanding business–society–nature relations merely through the lens of “stakeholder management” reduces the complexity of life to a managerial decision-making process, orchestrated by businesses. This is an extremely blinkered approach to understanding the world (Ehrnström-Fuentes & Böhm, 2022).

**Justice**

Once we have understood that there are many ontologies and ways of being in the world, it is important to recognize that not every human on this planet has the same destructive and extractive relationship to nature. In fact, the principal responsibility for causing climate change, ecosystem destruction, and species extinction lies with a wealthy minority.

As Mia Amor Mottley, Prime Minister of Barbados, said at the recent COP26 UN climate change conference in Glasgow, “The Central Banks of the wealthiest countries engaged in 25 trillion dollars of quantitative easing in 13 years; $9 trillion in 18 months. Had we used the $25trn to purchase bonds that financed the energy transition, we would be keeping within 1.5 degrees”. She made this remark in the context of the unequal distribution of the climate impacts already felt around the world. She asks: “Can there be peace and prosperity in one third of the world if two thirds are under siege and facing calamitous threats to their wellbeing?”.

The answer to that question is a resounding “No”, but the COVID-19 pandemic has shown that the wealthy countries continue to put themselves first. It is their economies and societies that come first; it is their economic interests that matter most. Yet what about their historical responsibility for causing climate change? More than 75% of cumulative greenhouse gas emissions since the start of the Industrial Revolution can be associated with countries in the Global North, i.e. the highly industrialized, wealthiest countries of the planet.

Yet the national borders of countries are artificial entities that, within a world of ecological crises, cannot do justice to the globality of the challenges we face. Many countries now have a very high Gini coefficient, meaning their distributions of wealth and income are extremely unequal. For example, many people in the coastal cities of China now live “first world” lives, whereas communities in the rural hinterlands struggle to feed themselves. In the richest country of the world, the United States of America, it is common to witness huge wealth gaps within even one neighbourhood. The general rule is—which does not seem to be universally understood—that the higher one’s income and wealth, the higher one’s consumption of resources and hence destructive impact on the planet.

The paradox is that those people, companies, and countries who shout loudest about being “green” are usually those with the highest environmental footprint. Germany, for example, is world-famous for its “energy transition” and championing of green business approaches, yet the country’s energy mix is still dominated by the burning of dirty coal and its automobile industry is still producing “gas guzzling” cars in their millions. Perhaps the most infamous example of such mismatch of talk and action is BP’s rebranding, in the mid-2000s, from British Petroleum to Beyond Petroleum, whereas the company only started very recently to invest more readily into renewable energy. Such mismatch needs to be called out and critiqued, framing it within a justice dimension. The most vulnerable people—particularly those already affected by climate change and other ecological crises—usually do not have access to the corporate communication channels, and thus fail to make themselves heard. Business ethics scholars have a moral duty to engage with this justice imbalance.

**Land**

The elites often live in cities and other urban environments, while most of the impact of global environmental change can be first and foremost witnessed by communities living close to the land and other rural landscapes. Business ethics and many other discourses are dominated by urban elites who only know about rural life from their holidays or maybe their second homes at weekends. While this is not to say that there is no poverty in urban ghettos—there clearly is—it is important to acknowledge that most Global South communities still live land-based existences, which are now under threat by droughts, storms, wildfires, and floods. In India, for example, most people make a marginal livelihood as subsistence farmers in geographies that are often not even connected to the electricity grid. Eighty per cent of the world’s poor live in rural areas (one-fifth of rural people live in extreme poverty), which is a rate that is four times higher than in urban geographies.

Yet it is the people on the land who produce the food we eat. Rural landscapes provide the natural backbone of urban existences. A world metropolis such as London could not exist without its vast rural hinterland, which extends all the way to Cornwall, the Ukraine, Southern Spain, and Kenya, for example, where some of the food it consumes is produced. As Jason Hickel tweeted recently: “For every $1 of aid the global South receives, they lose $30 through unequal exchange with the North. Poor countries are developing rich countries, not the other way around”. What he and his colleagues show in their recent paper (Hickel et al., 2022) is that it is a myth that “developing countries” need to be developed. Instead, through centuries of colonialism, imperialism, and resulting modes of unequal exchange, those countries that are poor today have transferred their wealth to the Global North. This transfer has often occurred in the form of raw materials and food stuff, which is dependent on the natural environment.

The urban elites now recognize the importance of the land for the future of a greener capitalism. “We” need land not only for growing food, but also to offset gigatons of carbon emissions and provide biodiversity corridors (in between the highspeed trains and motorways connecting the urban centres). The land is also important for people’s wellbeing, their relaxation, and replenishment (in between their Zoom calls). Natural flood defence mechanisms are being planned to save urban communities from the onslaught of climate change-induced storms. At the same time, the mining industry is busy repositioning itself as the provider of minerals required for the renewable energy transition. Everything from wind turbines to EV requires a vast amount of rare earths, copper, lithium and other minerals that need to be extracted from the Earth. The demands on the land are huge and will only increase in the coming decades, as the climate and ecological emergencies intensify.

**Degrowth**

If some of the above sentences sound somewhat sarcastic, then that is because they were intended in that way. The imaginaries of “green capitalism” are such that they depend on a set of contradictory assumptions. Land cannot be created out of thin air. It is a limited resource. The whole point of the very influential planetary boundaries research agenda is to show that planet Earth exists within certain boundaries that even *homo sapiens* cannot change. The limits of the planet are well understood, yet proponents of “green capitalism” still assume that it is possible to achieve a decoupling between economic growth and humanity’s destruction of the planet. That is, the basic understanding that lies behind any “green growth” (or clean growth, or green capitalism) approaches is that we can keep growing our economies in the exponential way we have since the 1950s if we find ways to do so without any (or manageable) environmental impact. This is a myth.

As the global community of degrowth scholars (e.g. D’Alisa et al., 2014) have shown for many years now, decoupling is not possible without questioning capitalism’s thirst for economic growth. Looking at the graphs of global greenhouse gas emissions over the past 20 years, they clearly show that reductions have only happened in deep, global economic crises, only then to bounce back very quickly. The formula still stands: the more economic activity, the more greenhouse gas emissions. Even if fossil fuels can be replaced by renewable energy, then there would still be a huge environmental footprint associated with that transition. Wind turbines need masses of land, they also require tons of minerals; and their current lifespan is typically 20–25 years and cannot be recycled at the moment. Also, let us be clear: globally, renewable energy has not yet displaced any fossil fuels at all. Wind and solar are simply supplying the growth in energy consumption worldwide, while fossil fuel usage is still growing too.

**Conclusion**

What this all means is that it will be inevitable to rethink capitalism’s logic of growth. The sooner we do this, the better the chance we have to address the global climate and ecological emergencies we currently face. The economic growth witnessed across the world since the 1950s is historically unprecedented, and this growth has only been possible through the overuse of fossil fuels. Yes, millions of people have been brought out of poverty as a result, but, at the same time, through mechanisms of ecologically unequal exchange, millions have also faced pollution, environmental degradation, and now climate change affects everyone on Earth. Business ethics—as a scholarly field and practice—needs to look these crises straight into the eye. There cannot be any pretence anymore. The challenges are urgent to address, and the Journal of Business Ethics must be at the forefront of tackling them.

## Climate Change and Business Ethics

**Boudewijn de Bruin**

**Introduction**

This article sketches ways in which business ethics should contribute to addressing the climate emergency. I consider some ways in which normative contributions to the debate on climate change and global warming have been defended, and how international thinking about environmental issues has moved from consequentialist to justice- and rights-based thinking. A recent case that came before the Hague District Court between a Dutch branch of Friends of the Earth, Milieudefensie, and Royal Dutch Shell (*Milieudefensie* v. *Royal Dutch Shell*), serves as an illustration of how human rights have taken centre stage in climate change litigation—and how business ethics has entered the courtroom. I use this case also to show where the contributions of our field lie: to think about consequences and principles, to include various stakeholders in our evaluations, and to conceptualize the responsibilities of business and politics.

Climate change is humanity’s biggest threat. When we contemplate the past 40 years of our Journal and seize the occasion to reflect on the next 40 years, we must take into account that what business will do in the years to come will determine the future of our planet for a very long time. Even in the most optimistic scenarios, our children will end up with a planet that is far too likely to be more hostile to human existence than ours was. In more pessimistic scenarios, the world as we know it may no longer be. Based on projections from the UN Department of Economic and Social Affairs, for instance, the most recent report issued by the Intergovernmental Panel on Climate Change states that around the turn of the century, an excess of more than nine million people will die from climate-related causes annually (Pörtner et al., 2022, pp. 62–63). Reflecting a justifiable sense of urgency and despair, the United Nations Environment Programme (n.d.) describes our condition as a “climate emergency”. In November 2019, the European Parliament similarly adopt ed a resolution to declare the “climate and environment emergency” (European Parliament resolution, 28 November 2019, 2019/2930(RSP)—and it was high time, given that the Oxford English Dictionary already includes a 1975 reference to “climate emergency”). We should, therefore, expect that 40 years from now our field’s current preoccupations—and the insights our field generates—will be evaluated to the degree they have helped us to find a way out of this emergency. So I ask here: What does business ethics have to offer to avert the climate emergency? (In passing, we might note that Nyberg et al. (2022) provide a recent article on business ethics and climate change in the special issue celebrating the 60th anniversary of a sister journal.)

But perhaps business ethics does have not too much to offer, one might think. The word “climate” appears 2749 times in our Journal, Springer’s search function tells us (March 2022). But only about a quarter of these hits are concerned with climate change in the way it is meant in this article. Our field has traditionally been vastly more interested in ethical or moral climate than in geological or meteorological climate change—and for good reasons, for we have thought, and keep thinking, that we can improve ethical decision making by changing the circumstances—the environment, the climate—in which people act. One may wonder if the immense array of extant business ethics theorizing developed over the past 40 years or so is anywhere useful under conditions of a climate emergency.

**Ethics**

A standard line of defence in this regard is that everyone who is engaged in decisions to do with climate change is doing ethics, and that doing ethics benefits from some professional guidance, for instance, about the so-called non-identity problem (e.g. see Setiya, 2017). Some have observed that, as our knowledge about climate change depends on projections grounded *inter alia* in computer simulations and econometric forecasting, we are constantly evaluating the plausibility and admissibility of such models of reasoning (Williamson, 2019). If we follow such a line of defence, to the extent that our field engages with epistemological and philosophy of science questions—which is happily more and more the case (de Bruin, 2013; see, for recent work in our Journal, Islam, 2022; Lamy, 2022)—our field might help avert the threat of the climate sceptic, for instance, by showing the dangers of social constructionist thinking the spell of which many still find hard to resist.

I do not think that the non-identity problem and the question of the social construction of reality are sufficiently connected to the daily concerns of people who are in the midst of environmental and climate change policy making. These issues are fairly ‘academic’. Yet this is not to say that our field is empty-handed. One can argue that claim in many ways. Given the space I have here, no fully fledged defence should be expected. I use a more tentative approach, and zoom in on a recent case in the Netherlands in which Royal Dutch Shell PLC, the oil refiner, was summoned to court by various environmental non-governmental organizations (“NGOs”), including Milieudefensie, the Dutch branch of Friends of the Earth, and Greenpeace Nederland. One can read this case as underscoring the increased relevance of business ethics to law, but also as showing us important issues to which we should contribute our knowledge and expertise. But, before turning to the case, I want to give very quick overview of how international normative thinking about the natural environment and climate change has evolved, and in particular how human rights have become more important.

**Law**

The story has been told quite often by international law scholars (Dupuy & Viñuales, 2018), but is worth recalling here (see also de Bruin, in press). It is, in terms of our field, a move from consequentialist thinking to thinking about rights. For some 50 years after the Second World War, international environmental disputes were resolved mainly on the basis of the no-harm principle. This was, for instance, the guiding principle used in the famous *Trail Smelter* case, in which a Canadian business harmed American forests and lands. This case articulated the principle that we must not use our lands in such a way that we harm our neighbour’s lands (*sic utere tuo alienum non laedas*—use your own property so as not to hurt other people’s: *United States* v. *Canada* (1938 and 1941) 3 RIAA 1905). Over the years, the no-harm principle became a cornerstone of international environmental disputes, ultimately making its appearance as Principle 21 of the Stockholm Declaration of 1972 (UN Doc. A/CONF 48/14/Rev.1). One may conceive of it as a kind of due diligence principle. It reflects, in my terminology, “state interest-based” reasoning (de Bruin, in press).

A further step was taken at the United Nations Conference on Environment and Development (also known as the “Earth Summit”), held in Rio de Janeiro in 1992. What happened there was that state interest-based thinking made room for what I call “shared interest-based” thinking (de Bruin, in press). The United Nations Framework Convention on Climate Change (UNFCCC) was established on the basis of the shared realization, expressed in its very first sentence, that “change in the Earth’s climate and its adverse effects are a *common* concern of humankind” (United Nations, 1771 UNTS 107, emphasis added). The Rio Declaration on Environment and Development, a further result to issue from the Earth Summit, introduced a number of principles in line with shared interest-based thinking, too, such as idea of common but differentiated responsibilities (Principle 7) and the precautionary principle (Principle 15) (UN Doc. A/CONF.151/26. Rev. 1).

More than 20 years later, the Paris Agreement took still another step (UN Doc. FCCC/CP/2015/L.9/Rev/1). Adopted in December 2015 at the Conference of the Parties to the UNFCCC, it linked shared interest-thinking to the notion of human rights:

Acknowledging that climate change is a common concern of humankind, Parties should, when taking action to address climate change, respect, promote and consider their respective obligations on human rights, the right to health, the rights of indigenous peoples, local communities, migrants, children, persons with disabilities and people in vulnerable situations and the right to development, as well as gender equality, empowerment of women and intergenerational equity. (Article 2, paragraph 1(a) Paris Agreement ^)^

**The Milieudefensie v. Royal Dutch Shell Climate Case**

On 5 April 2019, the Dutch NGO Milieudefensie (the Dutch branch of Friends of the Earth), other NGOs such as Greenpeace Nederland, and private individuals represented by Milieudefensie (‘Milieudefensie c.s.’) summoned Royal Dutch Shell PLC (‘RDS’) to reduce its greenhouse gas emissions. About two years later, on 26 May 2021, the Hague District Court ruled—to the surprise of many and the disappointment of some—that RDS should indeed reduce its emissions (Raval, 2021). RDS has an “obligation of result” concerning its “scope 1” emissions, which are the emissions with sources that RDS owns or controls itself; and it has “a significant best-efforts obligation” concerning its “scope 2” and “scope 3” emissions, which are emissions upstream in the supply chain and emissions downstream through the activities of end-users/consumers, respectively.

The court’s ruling is worth the read, and fortunately for those who, like me, want to use it in business ethics classes with international audiences, an English translation is available with its own European Case Law Identifier code (District Court The Hague 26 May 2021, ECLI:NL:RBDHA:2021:5339). It is worth reading, not only because it gives students a quick introduction into the more technical aspects of climate change and greenhouse emissions caused by companies such as RDS, but also because of its intriguing normative reasoning.

The idea in a nutshell is as follows. It centres round the Dutch civil law notion of tortious or “unlawful” act, which, very loosely put, captures *inter alia* situations in which one’s action or omission causes damage to someone else as a result of, loosely put, one’s failure to discharge some unwritten duty or standard of care (Macchi & Zeben, 2021). This leads in such cases to a very open norm; for where does that standard of care come from?

The trick of the Hague District Court in *Milieudefensie* v. *RDS* was to craft the unwritten standard of care using two ingredients: (i) the best climate science available as to the most effective way to mitigate and adapt to climate change (I’ll set this point aside here), together with (ii) the widespread international consensus that human rights should offer protection against the impacts of dangerous climate change and that companies must respect human rights (*Milieudefensie* v *RDS*, para 4.1.3).

Let’s pause and distinguish two things here. There is, first, the observation—also see my super quick discussion of the Paris Agreement above—that the dangerous consequences of climate change can or should somehow be characterized as human rights violations. Milieudefensie c.s. invokes the goals according to the Paris Agreement, and RDS has, outside and inside the courtroom, also committed itself to “support society in achieving the Paris Agreement goals” (para 2.5.20).

There is, secondly, an observation that, well, business ethics is getting more important. That companies must respect human rights is something business ethicists have long realized. But such instruments as the UN Guiding Principles, the UN Global Compact and the OECD Guidelines have often been set aside as “soft” law. It is, I think, characteristic of our times that soft law and business ethics are now entering the courtroom.

**Business Ethics**

But is the *Milieudefensie* v. *RDS* judgement to be applauded?

Coupling the threat of climate change and corporate human rights obligations the way the Hague District Court does is quite novel, to say the least. Smeehuijzen (2022) lists some of the pertinent questions this ruling invites us to ask: Doesn’t the court take the legislator’s seat, thereby endangering the separation of powers? Is this really something for a civil court to decide on, involving as it does so many interests? Can a whole multi-national group, with more than a thousand companies globally, be held liable? Do human rights have horizontal effect and should soft law be used this way? Is there sufficient causality to justify ascriptions of liability?

**Consequences and Principles**

Let me start with the last question. It’s not unique to *Milieudefensie* v. *RDS*, but I use this case to illustrate it. The big question is whether there is a causal link between RDS’s corporate policies and the alleged human rights infringements, or *modus tollens*, whether, if RDS were to reduce its scope 1, 2 and 3 emissions, this would indeed have the desired effect of mitigating climate change. The *Milieudefensie* v. *RDS* court evaluates this as follows:

RDS argues that the reduction obligation will have no effect, or even be counterproductive, because the place of the Shell group will be taken by competitors. Even if this were true, it will not benefit RDS. Due to the compelling interests which are served with the reduction obligation, this argument cannot justify assuming beforehand there is no need for RDS to not meet this obligation. It is also important here that each reduction of greenhouse gas emissions has a positive effect on countering dangerous climate change. After all, each reduction means that there is more room in the carbon budget. (para. 4.4.49)

This lands us straightforwardly in challenging ethical territory. It is to do with the responsibility we have for such large-scale events as climate change (de Bruin, 2018). It is to do with what business ethicists are quite good at. Over the years, we have developed ways to evaluate claims made by businesses to the effect that, if they stop certain ethically tainted operations, less scrupulous competitors will take over and substitute their activities, and the world will be worse off, or at least not better off. We have, that is, developed ways to weigh consequentialist considerations and rights- or justice-based considerations.

Adequate balancing of such considerations is more important than ever. The no-harm rule from above was plainly preoccupied with demonstrable damage done to directly affected interested parties, which requires very careful attention to the causal link between action (or omission) and the damage. Business ethics is specifically attuned to such questions, using insights from widely diverging normative approaches, realizing that managerial decision making often benefits from being confronted with a multitude of complementary normative tools.

**Shareholders, Employees, and Consumers**

Business ethics has also helped to spell out in a normatively appealing and practically useful manner the relevant interested parties to corporate decisions. This is stakeholder theory (Freeman et al., 2018). Using stakeholder theory as an analytical toolbox, we will immediately ask questions that the reasoning behind the *Milieudefense* v*. RDS* ruling seems to overlook: the fact that a normative evaluation of a corporation (more precisely, a group of companies such as RDS) must ultimately involve thinking about a large variety of people and organizations. Consider shareholders, among them the Netherlands pension fund ABP, who only in October 2021 announced their decision to divest from the fossil fuel industry entirely (Flood & Cumbo, 2021). Consider employees, about 10% of whom will be laid off during Project Reshape, which is part of RDS’s reorientation to a net-zero emissions economy (Raval, 2020). Consider managers, who are increasingly held to account for climate-related issues in administrative and civil courts, and potentially even in criminal courts.

Consider, finally, the “forgotten stakeholder”, RDS’s competition (Spence et al., 2001). In this case, one may benefit from being forgotten. RDS’s competitors have not (yet) been summoned by Milieudefensie c.s. (or anyone else), which led former Shell CEO Jeroen van der Veer (2021) to call this “discrimination” against Shell. He may have a point. The court, following Milieudefensie c.s., holds to the view that RDS is responsible for greenhouse gas emissions surpassing in quantity those of many states, including the Netherlands. But 85% of these emissions are in scope 3—that’s us, consumers, who cannot drive, or fly, or shop, or get their goods delivered without what RDS and other fossil fuel companies produce. When the human right to life is endangered by RDS’s emissions, shouldn’t we say that that right is first and foremost endangered by us? Shouldn’t Milieudefensie c.s. summon *us* as well?

**Business and/or Politics?**

Finally, whose responsibility is it really to take action? One potential complaint against *Milieudefensie* v*. RDS* is that it is a political responsibility rather than a business responsibility to develop and implement policies to mitigate and adapt to climate change (Smeehuijzen, 2022). The court is taking the seat of the legislator here. These are oft-asked questions in our field, related to corporate responsibility and corporate citizenship. My impression is that there is somewhat of a consensus to the effect that, yes, businesses do have some such responsibilities, but only provided the economic, political and legal environment is sufficiently aligned. If the playing field is very unlevel, then we may have to demand less.

Smeehuijzen (2022) makes us realize, however, that the *Milieudefensie* v. *RDS* judgement may have to be interpreted a bit differently: not as a straightforward question about how far a company’s responsibilities go, but rather as one about the roles and responsibilities of the judiciary in a situation of regulatory failure. This may go too far for many. But then it is important to see that the idea as such is not novel. When it became clear in the second half of the twentieth century that exposure to asbestos was linked to mesothelioma, regulators were unacceptably reticent to step in, with significant numbers of people dying as a result. It is clear that asbestos should have been banned much earlier, and civil courts accept that today by judging exposure to asbestos to be unlawful retrospectively. Similarly, it is conceivable that, when it comes to climate change, the judiciary, too, will have to step in and correct or mitigate regulatory failures. And indeed, some observers think that the *Milieudefensie* v. *RDS* case has started a new type of cases similar to those that, a few decades ago, brought the tobacco industry to its knees, at least in some jurisdictions (Brower & Raval, 2021).

**Conclusion**

I started this article with the observation that in 40 years from now, the success of our Journal and our field will largely be assessed in terms of our contributions to combating and mitigating climate change. That is why I asked the question what business ethics has to offer to avert the climate emergency. I showed how international normative thought about environmental issues has moved from consequentialist to human rights-based approaches, a claim illustrated by the *Milieudefensie* v. *Royal Dutch Shell* case. I discussed stakeholders (including the easily forgotten competitor). I looked into the division of labour between business and politics. And all this subsumed by the overarching theme of human rights. I showed that concerns that used to be seen as “soft” law now start obtaining binding force, which attests to the growing importance of business ethics. I argued that what our field has on offer is tools to reason about consequences and principles, tools to determine interested parties and the way interests must be weighed, and tools to evaluate the relation between politics and business. In sum, then, I have tried to show that our research agenda should lead us to exploit the large variety of methods and techniques that our enrich our field to help policymakers and businesses to avert the climate emergency.

## Consumer Ethics

**Deirdre Shaw, Michal Carrington, and Louise Hassan**

**Introduction**

The world is facing unprecedented challenges that impact social and ecological wellbeing on a global scale and implicate us as both as citizens and consumers. Current emission trends put us on course for climatic shifts that will have, and are already having, dramatic consequences for communities and environments around the world. Indeed, scientists are warning that the planet is now in the midst of the sixth mass extinction of species, and this extinction is driven by human activities. This, and the consequences it produces, is a critical issue for consumer ethics. In this commentary, we seek to stimulate discussion that looks forward and considers the principles and practices of our research and research outputs in the light of these urgent global challenges.

Current growth-focused approaches to production–consumption systems are unsustainable and are driving climate change, environmental degradation, and human misery. Yet, at the same time, material consumption of natural resources continues to increase. This includes consumer demand for cheap, “disposable” and short-lived items. The growth-oriented business models that underpin this over-consumption are often built on exploitation, are unsustainable and are, thus, no longer fit for purpose. Rather, new approaches to ecologically sustainable and socially just marketplace systems demand cultural change, a reconsideration of future interpretations of “consumer society”, a challenge to the dominant “growth logic” and stimulation of alternative ways to address our consumption needs. These issues are urgent, as is the demand for research that pushes the boundaries of current approaches to consumer ethics and presents contributions that advance our thinking both practically and theoretically.

**Understanding Consumer Ethics**

We are witnessing a rapid expansion of consumer ethics as a field of research, and what is viewed as “ethical” within this burgeoning stream of research encapsulates different expressions, concerns, and issues across individuals, groups and socio-spatial contexts. These expanding and diverse research approaches are resulting in complexity and heterogeneity in how we understand consumer ethics. This complexity is revealed in a recent review of the field by Carrington et al. (2021) published in the Journal of Business Ethics, who identify a number of avenues for future research, which we draw upon in this commentary.

First, research investigating the identity position of the consumer in consumer ethics has resulted in a plethora of terminology to describe the multi-dimensional ethically concerned consumer, including ethical consumer, green consumer, conscious consumer, political consumer, sustainable consumer, etc. While such terminology is at times used interchangeably, differences in nuance and usage provide a starting point from which to question what constitutes, and what influences, consumer ethics. Furthermore, consumers hold multiple roles and identities across, for example, gender, familial, national, political (etc.) categories, highlighting the complexities associated with multiple identity impacts on behaviour. Such identities will elicit differing emotional reactions and conflict when identity misalignments arise and consumption desires clash. Thus, how such identities relate to facilitate and inhibit consumer ethics is important to understanding the activation and relevancy of an ethics positioning in consumption contexts. Research is needed that places in a wider context how an ethical identity and ethical motives work alongside other activated motives and identities. Extending this perspective further, in addition to studying micro-individual perspectives and identity positions, it is important to consider the broader socio-economic, historic, political, and cultural milieu within which such consumption ethics identities are formed and situated. What is understood as “ethical” is graduated and context-dependent. Yet the extant literature is largely focused on relatively affluent Western consumers and has concentrated research on stimulating demand for “ethical” alternatives. Such an understanding is not reflective of the consumption lives and choices of consumers experiencing deprivation and the impacts of climate change in affluent societies, consumer refugees or those in the Global South.

Thus greater diversity in our conception of the consumer, their identity positions and consumption ethics, and attending to the contexts within which consumption ethics are formed, are necessary to avoid a narrow conception of what we mean by consumer ethics. In both Global North and South contexts, this also requires going beyond taking existing framings and applying them to such consumers, but rather taking the context as the starting point to critique theoretical frameworks developed from largely middle-class Western consumers, and to privilege different meanings of consumer ethics and expand the theoretical advancement of the field as a result. To offer an example, while calls to reduce levels of consumption, due to the impact on our planet, are important and necessary for affluent consumers, such action for those who are unable to consume enough to meet their survival needs is not appropriate.

Second, considering the diversity of consumer contexts also affords a critique of the limits of consumption alone as a form of ethical action. We must question assumptions that align marketplace purchases as inherently ethical without shining a critical lens on market structures and what they mean for different consumers. For example, what does consumer ethics look like in the context of a resource-constrained world; and, is ethical consumption reproducing the current capitalist market structural context that privileges individual and unrestrained consumption growth? This raises significant questions as to the boundaries and limits of consumer ethics under prevailing economic structures which are critical to guiding consumer behaviour, public policy and collective action. These questions of limits to our consumption due to decreases in planetary resources, alongside existing prevailing structures within the marketplace, are important, as it is often markets that determine what is “ethical” and curate the choices that are available for consumers. These questions critique the existence of consumer agency and the resultant responsibility and the extent to which consumers freely and rationally decide what constitutes an ethically superior choice. These questions also highlight the need to clearly understand the relationships between the factors that sustain current approaches to systems of production and consumption, and how consumers seek to make ethical judgements and take ethical action.

Third, this line of enquiry also highlights the uneven nature of allocation of resources across production and consumption systems globally—as different consumers have different experiences that impact their understanding and practice of consumer ethics. In seeking alternative approaches to consumption, we cannot separate consumption from production. It is thus vital to understand how approaches to ethical consumption are legitimated and normalized if we are to mobilize consumers to act as key stakeholders in the perpetuation or eradication of the many dimensions of the ongoing planetary crisis. This means understanding how consumer ethics is “regulated” both strategically and tactically by formal laws as well as by religious norms, customs and rituals, and how moral logics are ultimately structurally constructed, institutionalized and regulated by powerful institutional actors. Considering how we support the move to a more socially and environmentally just consumer ethics in a structural system that makes this ever more elusive is vital. This highlights the need for research that considers legitimacy and regulation, positioning the consumer as one of multiple institutional actors that recognizes differing cultural realms and multiple understandings and contradicts of what is ultimately regarded as “ethical”. Taking the circular economy as an example, the environmental impacts of a circular system, which requires waste collection, reprocessing and resultant output, are unclear—especially, in a global economy where few products are manufactured, purchased, disposed of and recycled in the same geographic location, leading to vast transfers of resources across the globe. It is vital that the circular economy is not viewed as a means to continue with current rates of production–consumption, since currently this is not sufficient to address the current climate crisis. Rather, volume of production and consumption must be addressed, because no amount of reusing or recycling will offset continuous industry growth. Further, stakeholders must be included, engaged, and given a voice globally. We need to identify smarter, slower, and more just systems of production–consumption that dismantle linearity and embed social justice and environmental protection.

**Conclusion**

In this commentary, we highlight the urgent need for new approaches and thinking to unpack how we understand socially just sustainability and the need to rethink current marketplace systems and revisit our understandings of logics of growth and consumer society. In doing so we need to acknowledge different consumer lived experiences, embedded in varying socio-economic, historic, political, and cultural contexts. While this is not designed to be a comprehensive wish list of submissions for the Consumer Ethics section of the Journal of Business Ethics, we do hope this serves to stimulate critique, discussion, and debate on the significant changes facing humanity today. As such, we very much welcome papers that respond to these questions and more. All perspectives and methodological approaches are welcomed.

## Inequality Re-examined: The Influence of the Capability Approach on Global and Corporate Governance

**Nelarine Cornelius**

**Introduction**

The Capability Approach or Capabilities Approach (CA), developed by Amartya Sen, a welfare economist and philosopher, and Martha Nussbaum, a classical philosopher, has attracted attention at the highest level, as has the work of Mahbub ul Haq, the Pakistani economist who led the team that developed the United Nations Human Development Report (HDR) and Human Development Index (UN HDI): issues of human dignity and development remained a core concern across their working lives. This followed ul Haq’s practical challenge of high office, as Minister of Finance in Pakistan, in the 1980s. Over the past 50 years, CA and its applications have been used by international institutes, national governments, international charities, non-governmental organizations (NGOs), multi-national corporations and, of course, academics, including those in the field of business ethics. The work of Sen and Nussbaum has attracted the greatest attention of business ethics scholars, whereas articles referencing the work of ul Haq are far fewer, despite the significant impact of ul Haq’s ideas.

**Amartya Sen: A Lifelong Quest to Address Inequalities**

I had a chance encounter with a recently published book by Amartya Sen, *Development as Freedom,* and his earlier work, *Inequality Re-examined*, which a colleague had recommended I read, after attending a talk given by Sen in Oxford. The books were a revelation, providing me with fresh insights and a clearer sense of the intellectual and practical difficulties of addressing inequality and social justice. Sen’s thinking was forged at the junction of theoretical, empirical, and practical reasoning. His work was a direct challenge not only to the normative concerns of welfare economists but also to, more generally, the question of how inequality could be understood differently.

When Amartya Sen was awarded a Nobel Prize for his work on CA (his prize was awarded for Welfare Economics), he donated an old bicycle to the Nobel Museum, the bicycle he used when he travelled around India to find out first-hand about the lives of the urban and rural poor. During the Bengal Famine, Sen gave school lessons to the children of the poor to improve their life chances. For his study of early years metrics in India, he travelled the country, making good use of rail and his trusty bicycle. Later, during his travels in India, he weighed babies and young children, spoke to families about the challenges they faced, and what their ambitions were for their children.

Sen’s work on populations and poverty. and in particular his work on the “100 million missing women” in the early 1990s, was also fundamental to shaping his thinking. Sen investigated the reasons for a shortfall in the number of women (relative to the expected number of women, given the usual birth and survival rates) in Asia, the Middle East, North Africa and Latin America relative to other countries and regions, measured through the male-to-female sex ratio. His econometric analysis identified the systematic, lifelong, gender disadvantage from before birth (with girls more likely to be aborted as “less valued”), and throughout their lives: “These numbers tell us, quietly, a terrible story of inequality and neglect leading to the excess mortality of women: regions and nations that do not allow women to achieve their full potential are more likely to underperform, in terms of their overall development and the pace of development, including economic development*”* (Sen, 1990).

**Neo-Aristotelian Ethics: The Capability/Capabilities Approach**

CA has its critics, concerned about what they argue to be CA’s universalist and individualistic, and asserted by some, neoliberal aspects. The UNDP commissioned a review on CA and Human Development in 2016, to reflect on the strengths of, and criticisms against, the CA. Osmani (2016) found that many of the criticisms were unfounded, and grounded in a misinterpretation of the philosophical rationale underpinning CA, as well as the normative concern, that “human development discourse rightly adopts the principle of ethical or normative individualism” (Osmani, 2016, p. 19), to guard against one community enjoying rights, while others have been denied them. A detailed account of CA is beyond the scope of this brief account. Important elements of CA are, first, that its principal philosophical antecedents are neo- Aristotelian, placing importance on an Aristotelian concept, *eudaemonia*, a life well lived, and what this would mean in modern times. This was the foundational idea that could be developed in terms of modern, globally relevant meanings quality of life human flourishing and human dignity. Posing the question, “equality of what?”, answers emerged from Sen’s detailed review of utilitarian, Kantian, and Rawlsian thinking. Sen acknowledges his debt to Rawls, but while Rawls’ focus is on the means of freedom (primary goods), Sen argues that standard-definition equality of opportunity is contentious, because “(1) the fundamental diversity of human beings, and (2) the existence and importance of various means (such as income and wealth) that do not fall within the purview of standardly defined ‘equality of opportunities’)” (1992, p. 7). Sen and Nussbaum held a common view of CA in terms of its central principles. Both were founder members and held the presidency of the *Human Development and Capability Association*, founded in 2004.

This includes the importance of the *extents of freedoms* and whether individuals and communities are free to pursue what they have reason to value, in order to fully function and flourish. The source of the extents of freedoms is through equality to exercise *capabilities*. These are *basic capabilities*, innate abilities that individuals possess, which through education, good health provision, etc., can be developed into *internal capabilities*, readiness to act within communities and society, which can be constrained or enabled through *combined capabilities*, internal capabilities, and readiness to act, operating within external conditions that may help or hinder internal capabilities (broadly, the socio-political and institutional environment). The array of capabilities an individual possesses is their capability set: the extent to which they can utilize their *capability set* within society is their realized functioning. *Instrumental freedoms* are those elements within a society that promote wellbeing and quality of life, and include political freedoms such as human rights, economic facilities, opportunities to utilize economic resources (e.g. wage earning, fair pay), social opportunities (facilities available across communities, such as education and health care), protective security (social safety nets to ensure life chances are maintained), and transparency guarantees (lucidity, transparency, and disclosure during disagreements, with clarity and equity of access to resolution and redress).

**Martha Nussbaum—Classical Philosophy Applied to Contemporary Social Challenges and Divergence From Sen**

The range of subjects that the philosopher Martha Nussbaum has addressed in her illustrious career is impressive, extending well beyond CA. Nussbaum has maintained an academic and advisory interest in women, sex and human development. Sen and Nussbaum collaborated in the early development of CA. Of note is their volume, *Women and Human Development*, published by UN WIDER, and Nussbaum’s works, *Sex and Social Justice* and *Creating Capabilities: The Human Development Approach.* Although the philosophical underpinning of both Sen’s and Nussbaum’s work is very similar, there are important points of departure.

CA had been pursued along a complementary but different path by Nussbaum, most noticeably with her development of a definitive list of human functioning capabilities: a universal set of normative capabilities that are generally protected by law. This is a position firmly rejected by Sen, regarding such a list as a potential “mausoleum”: what appears salient now may be viewed as outdated and time- or culture-bound eventually. In Ingrid Robeyns’ essay in *Feminist Economics*, in which she contrasts Sen’s and Nussbaum’s take on CA, she contends that the importance of social choice, and the mediating effects of voice, agency, context, community participation and social choice in policy development and distributive justice are crucial in distinguishing their contrasting approaches. Robeyns also notes that some countries, for example, Sweden, have developed their own lists, which contain some elements common to the Nussbaum list, but others which differ, as the context and rationale underpinning the list reflect imperatives for the Swedish state. The UN’s development of Sustainable Development Goals (SDG), whose roots lie in CA, were developed to determine its role in the twenty-first century, superseded its Millennium Development Goals. Another useful example is the UN’s Declaration of Human Rights, developed by a team led by Eleanor Roosevelt and established in 1948, four years after the establishment of the United Nations in 1945 after the end of the Second World War. In 2011, the “protect, respect, remedy” rationale that underpins the John Ruggie Framework on business and human rights, which became the UN Guiding Principles on Business and Human Rights, adds to the original UN Human Rights declaration by highlighting the ethical responsibilities of business.

**Mahbub ul Haq: Operationalization and Application of CA Thinking**

In his book *Reflections on Human Development,* Mahbub ul Haq made the following observations:

The real wealth of a nation is its people. And the purpose of development is to create an enabling environment for people to enjoy long, healthy, and creative lives. The simple but powerful truth is too often forgotten in the pursuit of material and financial wealth (1996, p. 15)*.*

This statement illustrates ul Haq’s move away from wealth and economic productivity as the basis for the evaluation of social wellbeing, but also creating a new view of the meaning of development: economic development does not benefit everybody. The assumed trickle-down from the wealthy to the poor could never be assured, ul Haq reasoned, since there was no guarantee that the rich would support spending on improving the health, education or other human development means of the poor. Further, national measures such as average earning per individual are insufficient to indicate an uplift in the lived experience of the poorest, given that factors such as literacy levels and life expectancy are fundamental to improving life chances.

Studying in Pakistan, the UK, and the US, ul Haq did not remain in academia to develop new ways of thinking about development: he worked for the Pakistan government as Minister of Finance. Frustrated that, in the aftermath of independence, 22 prominent families in Pakistan enjoying 66% of Pakistan’s economic expansion in the 1960s, he joined the World Bank before eventually working at the UNDP in 1989 with a team of economists, including Amartya Sen, who helped to shape the philosophical underpinnings of their work, with the CA. Ul Haq led the team that produced the UNDP’s first Human Development Report in 1990, and which has been produced annually thereafter. Ul Haq has also argued that many institutions, including the UN and the World Bank, needed to shift from a focus on national income to people-centred policies, and more broadly think different about global governance. Ul Haq also appreciated the importance of the right kind of measurement of human development, so that funds could be focused on institutions that supported the enhancement of quality of life. What resulted was the Human Development Index (HDI), which contains three core measures: economic prosperity, educational attainment, and life expectancy, as key to creating a more holistic view of national development. The UN Millennium Development Goals and current UN Sustainable Development Goals, both developed with the aim of creating a sharp reduction in numbers of people living in extreme poverty, have their roots in CA, and ul Haq’s view of the importance of considered but parsimonious measurement. Although the formulae underpinning the three main factors within the HDI was updated (in 2010), the HDI remains in use. Organizations that ul Haq was critical of in terms of their measures of development and need, such as the World Bank and International Monetary Fund, have adopted ul Haq’s thinking and, by implication, the underlying principles of CA.

**The Capabilities Approach, Corporate Social Responsibility, and Organizational and Global Governance**

Although CA was developed originally within the context of welfare and developmental economics, its potential for broader application has been seized upon by many. Along with colleagues, I developed ideas about the potential of CA to improve understanding of workplace inequality, as well as understanding better the challenges of community development and how organizations in the public, private, and social sectors could be evaluated in terms of their impact on the realized functioning of individuals and communities.

In the Journal of Business Ethics, there are about 100 articles that explore theoretically or empirically applications of CA, across most of the main subject areas within business and management, including, of course, economics as applied to business and markets. The application of CA in organizations is clearly of interest to academics and the range of topics covered is wide.

Although the range of issues covered is wide, and includes a long-standing interest in how the CA can create a new approach to business ethics theory and practice. Donaldson’s, 2001 article, ‘The ethical wealth of nations’, argues that.

Morality may create economic advantages for nations in ways that extend beyond the notion of an idealized market; and in order for ethics to drive economic advantage, ethical concepts must rise to the status of intrinsic value; and if claims for national ethical success factors are true, then nations should attend to the issue of moral education. (p. 25)

Donaldson draws on Rawls and in particular on Sen to make the case for the importance of ethical scrutiny of ideas, which have, for some time, been taken for granted among economics and business and management scholars. In addition to making the case for morality to sit the heart of ethical wealth creation, other scholars have scrutinized more closely the application of CA to corporate social responsibility and global governance, especially among the marginalized and the poor. Fia and Sacconi (2019) go further, using CA to propose news ways of developing rights-based social justice, including the responsibility of firms.

However, Kalfagianni (2014) expresses a note of caution, arguing that a CA analysis of private governance reveals that is likely to be insufficient when faced with global sustainability challenges and upward demand for goods (e.g. agribusiness), with supra-governmental structures and guidance being more likely to yield positive results. Indeed, in the field, Alamgir and Alakavuklar (2020) have used CA to investigate how women’s rights to formal employee recognition are neglected by global clothes manufacturers, despite claims of ethical procurement. Work on cross-sector partnerships (public, private and social sector) also highlights the challenges facing the social sector in attempting to work on behalf of local communities: although the social sector was most able to make community needs and voice visible, a CA perspective made it clearer that the creation of inclusive, voice-rich governance structures was imperative in order that community challenges were not neglected, and social organization influence was not submerged beneath the greater power and resources of the public and private sectors (Cornelius & Wallace, 2011).

Indeed, González-Cantón et al. (2019) consider that CA is gaining momentum as a theory of corporate responsibility and business ethics, and that the UN Guiding Principles have become a most important framework, with the authors developing proposals for rights-based CSR which has at its heart respect for human dignity. The importance of CA and supra-governance policies in relation to CSR and governance has gained momentum with the establishment of the UN Sustainable Development Goals and Ruggie’s Rights Framework. But is not just “big business” where CA is relevant.

The role of social organizations, vital for conveying needs “on the ground”, is also vital from a CA perspective. Alm and Guttormsen’s (2021) work on marginalized communities working for business corporations, and the challenges of “voice-rich” research, using case example of social organizations for which community voice is central to shaping their policies and operations, is a clear link to Sen’s work on the importance of public reason. Work on social enterprises (SE) also highlights the importance of clear CSR policies that ensure that they not only create social value and social goods in a responsible way, but that their internal CSR, behaviour towards employees and volunteers is also socially responsible also (Cornelius et al., 2008). SE created by traditionally disadvantaged groups can also be considered as lesser: lacking the capacity to create robust, sustainable social and public goods. A CA-based study of such organizations showed clearly that these assumptions, themselves grounded in a “disadvantaged-as-less-capable” perspective, was misguided (Wallace & Cornelius, 2010).

**Conclusion**

Time will tell whether businesses are simply engaging with these UN-based policies for PR purposes, or if more fundamental changes to business governance and social responsibility will result. The adoption of SDGs by accounting (e.g. ACCA Global, 2017) and global consultancy bodies, for example, are an indication of SDGs’ importance in governance and accountability policies. It is likely for the foreseeable future that CA will continue to be at the foundation of human development policy and, increasingly, CSR and corporate governance.

## Our Relationship to the Other: The Transformation of Business Ethic Conversations on (Cultural) Differences

**Laurence Romani**

**Introduction**

Contemplating 40 years of our journal and publications touching on what today we frame as the grand challenges of cultural understanding, justice, and equality, I perceive an important shift. Where ethics was once considered a peripheral aspect of business for many researchers dealing with cultural differences, it is now taking such a central place that, I will argue, it reframes our approach to the management of (cultural) differences.

This essay is an attempt to first broadly trace the development of business ethics concerns in connection with cultural differences, showing how international business interrogations are now much closer to the ones of diversity management. While the field of international business was originally concerned with expatriates acquiring knowledge and control over those seen as culturally different, typically during a foreign assignment, today’s global economy and cultural diversity of populations place the understanding of those culturally different at the core of most management practices. While cross-cultural management and diversity management were seen as separate fields, today they are closely intertwined, both interrogating our relationship to the one seen as different. In the second part of this essay, I elaborate on the heritage of the path taken by the field and how we need to manage this legacy. Finally, I consider emerging streams of study and how they are to place ethics at the core of our studies for cultural understanding, justice, and equality.

**Early Works: Showing the Cultural Components of Business Ethics**

With international economic expansion after World War II, managers discovered first-hand that business is done differently in various countries, not only due to local institutions and legislation, but also, as formulated by Hofstede’s (1980) study, due to different cultural dimensions. It became clear that management principles are cultural products – often from the US. This led to a fundamental question: if the way we do business is a cultural product, are business ethics practices also cultural? This interrogation animated debates for a couple of decades. Some scholars endeavoured to show the relativity of what is seen as ethical (relativism) and others worked on clarifying existing universal moral principles recognizable in each cultural environment (universalism).

The relativist argument has largely been investigated with the use of cultural dimension constructs, that is, a set of values comparable across cultural environments. These many studies have shown how culture influences ethical leadership and business ethics, for example, in decision making, ethical beliefs, ethical attitudes, drafting of codes of ethics, and, more recently, responsible corporate behaviour (see the review by Scholtens & Dam, 2007).

In parallel, some researchers embarked on providing emic descriptions stressing *how* this cultural influence is performed. They have elucidated local cultural interpretations of business ethics, and, in particular, the East and West divide (e.g. Resick et al., 2011). Aristotle and Confucius are contrasted in their understanding of virtue; *guanxi* is explained in view of Confucius principles; and Islamic ethics and its implications for business are investigated (e.g. Rice, 1999). In response to the cultural differences found, some engaged with devising global ethical leadership: a form of leadership that would be well received in many national cultures; and most progress was made with virtue ethics.

For other researchers, cultural variations must be the local expression of universal aspects of business ethics. Resisting the relativity of business ethics, they have made claims for a universalist approach, arguing that moral principles or virtues are universal in their approach and tenets across cultural variations (Demuijnck, 2015). This stream has made a significant contribution; however, it does not seem to have gathered much influence in cross-cultural ethics. It has generally been met with resistance, some arguing that preferences for universalism or relativism are cultural too.

Whether universal aspects of business ethics exist or not, an empirical problem still needs to be addressed: how do we deal with conflicting (cultural) views? The integrative social contracts theory (see Dunfee, 2006) provides a guidance. It suggests that certain moral principles are so fundamental to humanity that they are shared across cultural ethical environments. And, simultaneously, there are also so-called authentic norms, based upon the attitudes and behaviour of the members of a community, norms generated within a community’s moral free space. The theory articulates how certain ethical precepts are sometimes appropriate for different situations. It recognizes the variety in cultural values and preferences, refusing to impose a conception of the “good” and simultaneously recognizing transcultural moral understandings.

In retrospect, I believe that we can see how the relativist and universalist debate is linked to the early reality of international corporations’ operations. With expatriates, these companies expanded their operations across cultures and national environments that had distinct views on ethical business practice. They reflect the geopolitical order in place at the time: they often inform us about distinctions between the Western (implicit) norm of the expatriate and its encountered deviance. The problematization of cultural ethicality in also often done in terms of binaries: either around the relativism between the local or foreign reference, or the opposition between the local and the universal.

**Increased Mobility, and Resulting Diversity, Leads to New Business Ethics Concerns**

Today, work done in (virtual) multi-national teams (often with members located in different countries) is commonplace, providing a very different landscape for cross-cultural interactions than the one met by expatriates. Expatriates used to be in a bi-cultural situation: being the sole “foreigner” having to understand the cultural emic aspect of business ethics in their “host” environment. Now, in many international companies, employees often operate in teams composed of persons from different countries of origin, creating a multi-cultural environment.

What becomes more salient is thus the need to relate in ethical ways to the diversity of (cultural) differences, preferences, and (cultural) references met within a team; and how to work respectfully with this diversity. The increasing mobility of persons across countries is such that, today, even co-located teams are often composed of persons operating in a country other than their “home country”. Also called self-initiated expatriates, these persons have multiple cultural frameworks of reference, such as bi-cultural individuals or simply persons with a migration background. In addition, international corporations may cultivate a strong organizational culture to provide forms of cohesion around shared practices and values. In this situation, early comparative and emic studies provide limited knowledge. It is no longer possible to assume that a person who grew up in Singapore will adhere to ethical preferences previously identified by comparative studies between, for example, the US and Singapore. And this is more so if this person is working for, say, a Chinese corporation in Nigeria.

Business ethics in international business has thus transformed over the years and is now dealing with a situation that is familiar to another stream of research: diversity management. Managing responsibly the multiplicity of (cultural) perspectives present in a diverse group of employees has been a core ethical concern of inclusive diversity management (Pless & Maak, 2004). Contemporary international business and diversity management have now much in common: they both have a central interrogation regarding how to ethically relate to those who are seen as (culturally) different. Cross-cultural business ethics was originally seen by many as being a side-topic, a problem that only some expatriates would encounter in countries where corruption was perceived to be high. Today, I argue, ethics is at the core of (international) management because it touches on how we engage with the diversity present in every workforce. The question of “how can we relate to differences in a respectful way?” is now core to both diversity and international management.

The reality of current international business operations also changes the scholarly questions we are posing. When we previously wondered whether a leader could be perceived as ethical across various cultural environments, we now investigate the possibility of ethical leadership in a global environment. For example, how can ethical leadership of multi-cultural teams be theorized? The topic of responsible leadership, for example, takes a much more central place in contemporary international business, precisely because it relates to multiple stakeholders with divergent (cultural) preferences.

**Managing our Heritage and Thinking Along New Lines**

Many works submitted to the Journal of Business Ethics continue the heritage of early comparative studies: they document how (national) cultural values influence what is perceived to be ethical. These works often meet the challenge of providing a significant and distinctive contribution, because we already know that ethics has cultural components. In addition, they often use cultural dimension frameworks. These frameworks were needed to provide tangible evidence of the cultural components of business ethics, but we also recognize today their important limitations. They assume a strong homogeneous view within nations, when we know that such homogeneity cannot be taken for granted, nor can we assume that nationals from this country will adhere to these principles, either at home or when operating in a different cultural environment.

These works often also prolong a cultural imperialist view on ethics, showing quite often how non-Western practices differ from what many consider good ethical practices in the Western world. While critical and post-colonial studies have stressed the colonial legacy of international management, the literature on business ethics has yet to interrogate the colonial heritage of our knowledge production. We need to consider the potential epistemic violence of marginalization done against indigenous knowledge development from the Global South. This can be done, for example, with epistemic healing: bringing to the centre of our discussion in business ethics the knowledge traditions that were excluded and made peripheral (see Khan & Naguib, 2019).

Besides studies about Confucian ethics, few works engage today in an in-depth emic presentation of cultural construction of ethicality. These works face a delicate task: to provide what can be called an authentic presentation, that is, a presentation that is not linked to (etic) comparative cultural dimensions but to the understanding(s) attached to the relevant community, and, simultaneously, making this presentation understandable to persons outside this community. Another challenge is to present non-Western philosophical traditions in a style that stays away from Orientalism, that is, presenting them not solely in view of their similarity or difference to well-established Western perspectives. Yet another challenge is to present the inherent diversity of these emic understandings, and not reduce this plurality to a narrow definition and a single definition (e.g. ‘Ubuntu’). When they successfully address these challenges, such emic studies provide a rich understanding of the (cultural) construction of ethicality (see Lutz, 2009) and have the potential to contribute to shape the possible forms of global ethical management, that is, the ethical management of (cultural) differences.

So, rather than prolonging a cross-country comparative view on business ethics, I believe that contemporary concerns that place our relationship to another at the centre of our interrogation of business ethics are a promising avenue. This enables us to address the limits of existing representational practices, in which viewpoints from, for example, non-Western populations risk being distorted. In addition, the simplification of those seen as different as being solely a cultural product becomes untenable. The necessity to approach them at the crossroad of several constructing experiences and identities leads to long overdue intersectional approaches in our understanding of the others. In sum, a focus on our relationship to multiple stakeholders, or simply the Other, will place them at a central place in our theorizing.

With a shift from comparative descriptive studies to works that focus on the understanding of our ethical relationship to the Other, we can further enrich our philosophical and theoretical inspirations. Responsible leadership and its relation to virtue ethics seems to be a path to explore. It can continue the knowledge achieved in (global) ethical leadership and other value-centred views on leadership, yet add a strong relational component to multiple stakeholders, or simply put, to the Other. Another possible path to explore the self-other-world relationship is proposed by Janssens and Steyaert (2012) through the multiple understandings of cosmopolitanism. Cultural cosmopolitanism, for example, adopts an open-mindedness to differences found in the plurality of cultures, with an ethical stance that expresses a responsibility to know and represent the Other in a non-hierarchical way. This provides a novel theoretical inspiration for the study of inclusion, translation, and hybridity that take place in international management.

In addition, the synergies that can be found between diversity management and contemporary international management open the way for new theoretical inspirations. In international management, the tensions between universalism and particularism have built principally on moral universalism, consequentialism, and utilitarianism, as well as virtue ethics. The knowledge and approaches developed in diversity management provide complementary as well as new inspirations, especially with works touching on inclusion. In theorizing inclusion, utilitarianism appears to be resisted, for example, but the multiple facets of the ethics of justice are explored (Jammaers, 2022). This has not yet been the case in research on ethics in international management. Virtue ethics is a strong inspiration for thinking inclusion, as well as ethics of care, but my impression is that the latter is practically absent from business ethics debates in international management—along with feminist views on ethics (see, e.g. Johansson & Wickström, 2022). Studies also point to how individualist and so-called masculine logics come in opposition to more collective ones in contemporary Western approaches to inclusion. In sum, starting a dialogue between the works and inspirations from diversity management and international management is a potential avenue for new insights in our discussions on business ethics.

**Conclusion**

Concluding this short essay, I realize how much progress we have made in our conversation on (cultural) differences and business ethics, but also how important this conversation is today. Our concerns have shifted from rejecting a parochial view on business ethics by documenting and measuring the cultural components of ethicality, to the interrogation of how to respect (cultural) diversity and how to work on its inclusion (Janssens & Steyaert, 2012). The grand challenges of cultural understanding, justice, and equality reflect the world we want to live in. They are particularly relevant today, as I am writing these lines at a time when Russian military forces are thrown into a war on Ukraine, shaking our taken-for-granted views that respect of the Other is commonly seen as desirable. Rather, engaging in an ethical relationship to the Other is a choice that we are making, and this in view of what we believe are possibilities for sustainable societies. This is a choice that might increasingly be questioned or challenged, and this is why we need to continue this conversation to further explore the premises, tenets, and implications of, and alternatives to, this preference.

## What Can Feminism do for Stakeholder Theory (and Business Ethics)?

**Charlotte Karam and Michelle Greenwood** 2

**Introduction**

The year that the Journal of Business Ethics was founded, 1982, was just one year after the United Nations’ Convention on the Elimination of All Forms of Discrimination against Women (CEDAW) entered into force as an international treaty. The Convention serves both as an international bill of rights for women, and as an agenda for action, placing a set of legal obligations on each member state to ensure that it does not discriminate through its own action, and that it puts in place mechanisms to eliminate discrimination by private individuals and organizations. To date, of the 193 UN member states, 187 states (excluding the United States, Tonga, Sudan, Somalia, Palau, the Holy See, and Iran: United Nations, n.d.) have ratified or acceded to the CEDAW.

CEDAW was a major milestone led by the UN Commission on the Status of Women, marking over 30 years of stakeholder mobilizing and deliberations. Even with such efforts, women’s inequality remains an unevenly pervasive problem across geography, culture, and vocation, with our own discipline being no exception. The complexities of this pervasive problem are broad and deep. Understanding such complexity requires the unpacking of how inequality is systematically structured in economic, social, political life and the ways in which it is associated with recurrent patterns of unequal distributions of opportunities, rewards, wealth, services, goods, punishments, and, of course, most poignantly, power.

Centring of power in our consideration of women’s and other marginalized stakeholder experiences, casts attention on questions as to how different identity categories (e.g. sexual, class, caste, race, ethnicity, religion, location, ability, etc.) interact to (re)produce and transform relations in the context of individuals’ social and material realities. Understanding this interaction is of key importance, because it allows for the examination of how categories of identity are intertwined and mutually constitutive. Examination of such interactions emphasizes that patterns of inequality are often experienced as complex forms of oppression which are inherently tied to intersectional identities and thus cannot be disentangled (Crenshaw, 1991). This focus on intersectionality is helpful in understanding a broad range of experiences that can be analytically useful to unpack stakeholder dynamics, to theorize stakeholder identity, and, additionally, to trace power dynamics as a property of institutional and organizational structures and disciplinary discourses not often considered when approaching ethical analysis of business and business-related phenomena.

In this commentary, we advance the question of *what can feminism do for stakeholder theory.* It is our contention that, through bringing the work of feminist scholarship to stakeholder theory, greater attention would be afforded to examining the vast range of interacting and interlocking systems of identity and inequality that shape experiences of women and other historically marginalized individuals within and across different stakeholder groups. As will be argued in the pages that follow, applying a feminist lens to stakeholder theory brings to light the oft-hidden power dynamic relating to forms of oppression and the day-to-day realities of different groups.

**The Problem for Business Ethics**

Issues around inequality dominate social, political, and economic debate globally and locally. Whether it is the exodus of women from a war zone, the rise of intimate partner violence during a pandemic, or the rape of a political staffer by a colleague in the Australian Parliament House. Despite important work bringing questions about gender and feminist thinking to corporate social responsibility and business ethics (Grosser & Moon, 2019; Karam & Jamali, 2017; Machold et al., 2008; Prieto-Carrón, 2008), this important area of analysis remains marginalized within the broader discipline. Furthermore, stakeholder theory, as a leading theory in these fields, is similarly limited in its engagement. This is a problem, not just with regard to ignorance of these vital matters, but because a feminist analysis contributes more broadly to debates around power, voice and the manifestation of forms of oppression.

Debates in stakeholder theory have been centralized around three fundamental questions: (1) What is a stake? (2) Who is a stakeholder? and (3) What is the nature of the organization-stakeholder relationship? From the outset, the conceptualization of *what is a stake* has marked stakeholder theory as departing from traditional theories of the firm. While there are a wide variety of responses to this question within the theory, characterized by Kaler (2002) as either focused on moral claims or strategic influence, there is important agreement that a focus on capital or property, as narrowly conceived by shareholder theory, is inadequate to explain the ways in which real firms interact with those in their environment and create value. The corresponding question of *who is a stakeholder* tends to be foreclosed by using a list of functionally based roles (e.g. employees, customers, financiers, community, and so on), shifting the debate to deciding which of these stakeholders are in or out. Are, for example, trade unions or competitors to be considered legitimate stakeholders, and why or why not? This has been most readily resolved with the idea that there are categories of stakeholder—primary, definitional stakeholders, and secondary, indirect stakeholders—and that these vary from company to company and/or industry to industry. Related inquiry into *what is the nature of the organization-stakeholder relationship* often assumes a central organization is in interaction with distinct and distant stakeholders, who may or may not be in interaction with each other.

Underlying these responses to the three questions are a number of implicit, problematic assumptions. First, that those who are identified within a stakeholder group share characteristics and interests to the extent that they can be treated as equivalent, and that the group is somehow internally homogenous. Second, that a stakeholder group is a distinct entity to the degree that it can be considered separate from the organization, from other stakeholders, and from broader structural considerations. Third, and arising from the previous two, that transactions between an organization and its stakeholders will be efficient and ethical if based on reciprocity. We believe that this way of understanding stakeholder relationships is overly simplistic, stripped from any substantive acknowledgement of power dynamics, and cannot therefore account for fluid and complex relational issues, in particular issues of inequality that relate to oppression and are inherently tied to intersectional identities, such as those raised early in this commentary. Hence we challenge each of these assumptions by employing feminist analysis.

**A Feminist Response**

Despite its marginalization in business ethics and organizational studies, feminist theory has a long history and covers a broad and often divergent range of positions (see Calas & Smircich, 2006). Indeed, early work on the influence of feminism in stakeholder theory stands here in good stead (e.g. Buchholz & Rosenthal, 2005; Derry, 2012). Notwithstanding the diversity of feminist positions, several analytic heuristics can be identified that are broadly agreed. These heuristics can be used to further flesh out the underlying complexities of stakeholder theory’s three fundamental questions noted earlier. We discuss each in turn.

**What is at stake and for whom?** Adopting a feminist lens leads us to attend to lived experience of particular individuals or groups and to not assume that the universal experience holds for all. What is a stake—whether in conception, quantity or kind—is different for different stakeholders, and these differences are inherently tied to context and identities. As noted in the opening text of this commentary, categories of identity are intertwined and mutually constitutive (Crenshaw, 1991), and adopting an intersectional lens assists in tracing power dynamics as a property of institutional and organizational structures and disciplinary discourses that ultimately shape individual experiences. The interconnected nature of identities as they apply to a given individual or group work to set the basis of complex experiences of the overlapping, interdependent systems discrimination or disadvantage. Adopting an intersectional lens is very important to understand, not only the intricacies of lived experiences of stakeholders, but also how different organizational and institutional systems create and perpetuate social structures that shape this experience (e.g. Bondy & Charles, 2018).

Paying close attention to the lived experience of intersecting identities surrounding what is at stake, and for whom, with the goal of interrogating and disrupting unequal power relations and outcomes, is a key heuristic in feminist inquiry into problems and solutions. There are often hidden assumptions about what is at stake and for whom. These assumptions must be examined, particularly with regard to how those assumptions and theories might be complicit with forces of domination, oppression, and social exclusion in our examination of the lived experiences in the network of stakeholders.

**Who is a stakeholder and in what ways are stakeholders interconnected?** Many feminist schools of thought call for recognition of ourselves in the world as interconnected with others and to not assume ourselves as a separate entity with independent agency and interests. Given the ontological commitments of feminist theory, the response to this second question is that the holding of a stake can be understood as arising from engagements with focus on how one comes to be (i.e. becoming) a stakeholder, rather than a solid state of being stakeholder or having a stake. Stakeholder identities and subject positions are not seen as bestowed or predetermined, but rather constituted through discourses, materialities and practices (Greenwood & Mir, 2019). For example, the wearing of masks in service settings may limit a customer’s experiences of the emotional concerns of a service provider. In this way, therefore, the (re)production and transformation of relations are intimately intertwined with contextual dynamics and therefore the interconnection between individuals’ social and material realities.

Of further importance to build into our considerations of who is a stakeholder is ourselves as stakeholders, bringing a critical consciousness of our own social locations as researchers. The constructive, corrective value of a reflexive stance afforded with this step introduces the methodological principle of epistemic accountability (Anderson, 2004) and also makes a particularly compelling case for the importance of needing to be reflexive about our positions vis-à-vis knowledge, and our consideration of the ways in which our epistemic practices may perpetuate injustice (Fricker, 2007) within our understandings of who is a stakeholder.

**What is the nature of the organization-stakeholder relationship and what potentialities do organization-stakeholder relationships hold?** Arising from the previous two questions, feminist work brings forward an understanding of stakeholder exchanges as based on interconnection and relationality rather than separateness and exchange. This, therefore, moves stakeholder thinking beyond assuming reciprocal transactions as the hallmark of efficient and ethical co-operative arrangements. Rather than understand the relationship as the exchange between two bounded discrete entities, a feminist reading would hold that they are interconnected, such that one does not exist without the other. Furthermore, and with a feminist understanding of the broader nature of the stakeholder relationship, inherent questions concerning the possibilities for transformation and the processes for changing social relations arise.

Opening up stakeholder theory to these feminist positions on responsible relationality allows for deeper exploration of the ethico-political dimensions and of forms of feminist resistance that has the goals of social justice and equality. In what ways does it foster the organization of and solidarity and empathy towards more dignified and mutually beneficial stakeholder relationships? For example, the question of what ways might feminist resistance augment the organization-stakeholder relationship and move away from perpetuating inequalities can been addressed in various situated contexts (e.g. in the Middle East: Aldossari & Calvard, 2021).

Adopting such a relational lens gives particular attention to local realities and commonalities between local and global, so that we can build solidarity across borders, and so that we can build stakeholder relationships that better contribute to a more socially just world. In this vein, Mohanty (2003: 505) coins the relational term “common difference” and asserts the following:

In knowing differences and particularities, we can better see the connections and commonalities because no border or boundary is ever complete or rigidly determining. The challenge is to see how differences allow us to explain the connections and border crossings better and more accurately, how specifying difference allows us to theorize universal concerns more fully.

**Possible Futures**

Feminist-inspired stakeholder theory can contribute to current explorations in the *micro foundations of organizational-level* phenomenon such as corporate social responsibility. For example, Ozkazanc‑Pan (2019) argues, using the case of the Rana Plaza collapse in Bangladesh, that conceptualizing CSR with gender brings attention to the particular and embodied Other and focuses on the relationships underpinning responsibility. Within this context, the author traces the ways in which adopting a feminist lens when analysing CSR programmes helps to highlight the importance of understanding how employees are subject to different socio-economic conditions and experiences across intersectional relations of difference, and that such an understanding is vital for discussion in business ethics and corporate responsibility.

In juxtaposition, calls for more *macro analysis of individual-level phenomenon* would also be informed by this perspective. Currently, the topic of intimate partner violence (IPV) is being explored in business ethics research, building on research in sexual harassment, bullying, and discrimination. Rather than take the IPV victim as the unit of analysis, feminist analysis refocuses the attention to, for example, asking how (and in what ways) might victims benefit from flexible work arrangements. Similarly, feminist analysis might also examine institutional practices and policies that may unintentionally perpetuate or enable control or violence by abusive partners (Scott, forthcoming). Indeed, a feminist approach to stakeholder theory would open inquiry to take in a broad range of stakeholders and ways in which organization-stakeholder engagement might contribute to or mitigate gender-based violence.

These types of explorations call for deeper methodological commitments. Inquiries that demand more fine-grained, situated, and thick data require research design and methods that allow for the collection and analysis of such data. For example, if there is a question regarding the (on the ground experience of) reporting sexual harassment, rather than survey human resource managers about their sexual harassment policies, researchers might interview these practitioners to inquire what tensions and contradictions they experience when formulating and implementing such policies. Indeed, rather than interview the HR practitioners about the tension and contradictions of sexual harassment policies, researchers might attend HR policy meetings, attend management training sessions, interview union officials, interview workers, observe counselling sessions, and so on. Furthermore, in participating in such an inquiry a researcher needs to not only reflect on the good or harm potentially arising from the design and implementation of sexual harassment policies but on the good or harm potentially arising from their own research intervention. Such research design moves the inquiry beyond the HR practitioner-worker dyad and allows for the exploration of the phenomenon across a broad range of stakeholders (including the researcher themselves). Furthermore, positioning the problem at both organizational level (e.g. structures, policies, and processes of reporting) and individual level (e.g. who, when, how to report and respond) opens up questions around power, participation and silencing.

New areas of research and types of questions open up with feminist-inspired stakeholder theory. Take as an example the question of what the status of technology is in stakeholder theory. A feminist understanding of the porosity of entities could lead to not only rethinking stakeholder relationships between humans, but also with non-humans. The notion of non-humans as stakeholders was debated with regard to the natural environment in the early days of the development of the theory, with only limited attention paid to non-humans as stakeholders since then (Tallberg et al., 2021). Despite the material turn in organization studies and, to a lesser extent, business ethics, the notion of technology as relationally agentic has been largely ignored (den Hond & Moser, 2022). This is not to say that technology has not been debated with regard to CSR, business ethics and stakeholders, but there is minimal evidence of serious consideration within stakeholder theory of the status of non-humans, such as artificial intelligences, let alone as stakeholders in and of themselves (i.e. having agency and interests). Such lines of inquiry have been exemplified through the work of technofeminists (e.g. Wajcman, 2006), who delineate the technology-related mechanisms through which oppressive gendered social processes and interactions are maintained and propagated. Understanding humans through a feminist lens highlights people as interconnected, not just with other humans but with and through the social and physical world; it enables exploration of the ontological status of technology and how it might problematize the core questions of what is a stake, who is a stakeholder, and how should the organization-stakeholder relationship be organized.

**Conclusion**

In sum, we posit that feminist analysis in business ethics and CSR goes way beyond “women on boards” or even the feminism section of the journal. Feminist-inspired theories, methods, and positionality can be brought to many spheres of our discipline, including, not least of all, stakeholder theory. Bringing feminism to stakeholder theory can broaden and deepen its capacity to be more nuanced, responsive, and transformative.

The Journal of Business Ethics has been commended for leadership in feminist scholarship (Bell et al., 2020) and in the launching of a dedicated Feminism(s) and Business Ethics section in 2018. However, we take only slight comfort in these small steps forward. Thus, we choose this 40th anniversary commemorative occasion to advocate for some heavy lifting to be undertaken by business ethics scholars, far sooner than in the next 40 years. We cannot wait that long.

## Footnotes

1 All three authors of Reimagining Grand Challenges as Grand Ethical Challenges: From Grandiose to Humble Solutions have contributed equally and should be considered as shared first authors.

2 Many of the ideas in this commentary have germinated with our co-authors. Thus, we acknowledge Ed Freeman, Harry Van Buren, Raza Mir, and Fida Afiouni.
